# Osteoconductive Potential of Barrier NanoSiO_2_ PLGA Membranes Functionalized by Plasma Enhanced Chemical Vapour Deposition

**DOI:** 10.1155/2014/253590

**Published:** 2014-05-04

**Authors:** Antonia Terriza, Jose I. Vilches-Pérez, Emilio de la Orden, Francisco Yubero, Juan L. Gonzalez-Caballero, Agustin R. González-Elipe, José Vilches, Mercedes Salido

**Affiliations:** ^1^Nanotechnology on Surfaces Laboratory, Instituto de Ciencia de Materiales de Sevilla (CSIC), Universidad de Sevilla, Avenida Américo Vespucio 49, 41092 Seville, Spain; ^2^Facultad de Medicina, Servicios Centrales de Investigación en Ciencias de la Salud, University of Cadiz, Dr. Marañon 3, 11002 Cadiz, Spain

## Abstract

The possibility of tailoring membrane surfaces with osteoconductive potential, in particular in biodegradable devices, to create modified biomaterials that stimulate osteoblast response should make them more suitable for clinical use, hopefully enhancing bone regeneration. Bioactive inorganic materials, such as silica, have been suggested to improve the bioactivity of synthetic biopolymers. An in vitro study on HOB human osteoblasts was performed to assess biocompatibility and bioactivity of SiO_2_ functionalized poly(lactide-co-glycolide) (PLGA) membranes, prior to clinical use. A 15 nm SiO_2_ layer was deposited by plasma enhanced chemical vapour deposition (PECVD), onto a resorbable PLGA membrane. Samples were characterized by X-ray photoelectron spectroscopy, atomic force microscopy, scanning electron microscopy, and infrared spectroscopy (FT-IR). HOB cells were seeded on sterilized test surfaces where cell morphology, spreading, actin cytoskeletal organization, and focal adhesion expression were assessed. As proved by the FT-IR analysis of samples, the deposition by PECVD of the SiO_2_ onto the PLGA membrane did not alter the composition and other characteristics of the organic membrane. A temporal and spatial reorganization of cytoskeleton and focal adhesions and morphological changes in response to SiO_2_ nanolayer were identified in our model. The novedous SiO_2_ deposition method is compatible with the standard sterilization protocols and reveals as a valuable tool to increase bioactivity of resorbable PLGA membranes.

## 1. Introduction

Cell-biomaterial interaction is a major challenge for tissue engineering, since not only the physic-mechanic-chemical features of the natural extracellular matrix must be mimicked, but also a successful cell to biomaterial adhesion should be achieved in the shortest time. Cell adhesion is critical in the first stages of the tissue engineering methodology where adhesion is the basis and the key process that allows other important cell activities such as migration and growth. In the fields of tissue engineering and guided bone regeneration (GBR), an appropriate cell culture scaffold is required to enhance cell attachment, proliferation, and differentiation in vitro, as cells interact with native or topographically and chemically tailored structures in many ways [1–5].

The GBR therapeutic approach is based on the concept that regeneration of osseous defects is attainable via the application of occlusive membranes, resorbable or nonresorbable, which mechanically exclude nonosteogenic cell populations from the surrounding soft tissues. Nowadays GBR is subject of growing interest due to the increasing needs for permanent, temporary, or biodegradable orthopaedic devices designed for bone repair and regeneration [6–11].

A variety of polymeric bioresorbable GBR membranes that permit single-step procedures, thus reducing patient discomfort and costs, and potential surgical complications are currently available. Polymeric coatings for biomedical devices are especially interesting due to the diversity of their chemical and physical properties and natural biopolymers appear promising as biomimetic coatings, as a result of their similarity to human tissues. Although these membranes are biocompatible, nontoxic, or immunogenic and permit mechanical support during bone formation, they do not actually possess bioactive properties to induce osteogenesis [6, 9, 12–14].

Poly(lactide-co-glycolide) (PLGA) is among the few synthetic polymers approved for human clinical use due to its biocompatibility, controllable degradability, and relatively good processability and has been used in the design of scaffolds for bone tissue engineering and for drug delivery purposes [15, 16]. However, the polymer itself  is quite bioinert and does not elicit a significant biological response in bone cells [8, 16].

The physicochemical properties of the material at the cell-material interface are decisive for the cell-material interaction, due to its interfacial role between the material and the host tissue, and are able to trigger a wide variety of processes, from the initial inflammatory reaction to ultimate tissue remodelling [13, 17]. Although the bulk properties of materials are important for the overall properties, especially for mechanical strength, a topographical modification of the cell-substrate interface is of utmost importance. Thus, the possibility of tailoring membrane surfaces, in particular in biodegradable devices with bioactive properties, to create modified biomaterials that mimic natural bone extracellular matrix (ECM) and stimulate osteoblast response should make them more suitable for clinical use, hopefully enhancing bone regeneration in the near future [1, 2, 18].

Bioactive inorganic materials, such as silica granules, have been suggested to improve the bioactivity of synthetic biopolymers in promoting the osteogenic performance of osteoblast-like cells. It has been strongly suggested that silicon/silica is functionally active during bone formation with a close relationship between silicate and calcium deposition. Silica is a low cost and biocompatible inorganic material having excellent chemical stability with great commercial value and ideal for tailoring surface topographies [19–24].

We have designed an in vitro model in order to assess normal human osteoblasts, HOB, response to a series of SiO_2_ functionalized PLGA membranes intended to be used as barrier membranes for bone guided regeneration.

For the synthesis of the PLGA foils we have followed classical synthesis procedures based on the solution/evaporation method [25–27]. A problem encountered by the surface functionalization of these membranes by classical sol-gel or similar wet chemical routes is the degradation of the PLGA when it is exposed to liquid media or treated at relatively high temperatures [28–34]. To avoid these problems, we have followed a new approach consisting of the deposition at room temperature by plasma enhanced chemical vapour deposition (PECVD) of a very thin layer of SiO_2_ on the surface of the PLGA membranes (SiO_2_/PLGA) [35], in order to achieve a strict control of the deposition conditions without inducing any damage in the PLGA that could efficiently promote the development of bone regenerating cells.

## 2. Material and Methods

### 2.1. Cell Culture

HOB human osteoblasts were incubated in Osteoblast Growing Medium with 10% foetal calf serum at 37° and 5% CO_2_ until the experiments were started. HOB cells did not exceed ten population doublings. Test surfaces were sterilized under ultraviolet (u.v.) light for 20 min each side, in a laminar flow chamber prior to cell seeding. HOB osteoblasts were seeded at a density of 5000 cells/cm^2^ on test surfaces, that is, bare PLGA or SiO_2_/PLGA membranes, and immunolabelled after 24, 48, and 72 hours. HOB osteoblasts, media, and sera were obtained from Promocell (Heidelberg, Germany).

### 2.2. PLGA Membranes

The PLGA membranes used as substrates were prepared from a 1.5 wt% PLGA dichloromethane solution by evaporation of the solvent on a teflon plate. The size of the membranes was 15 × 15 mm^2^. The thickness of the membranes was of the order of 50 microns.

### 2.3. Deposition and Characterization of SiO_2_ Thin Film Layers

SiO_2_ was deposited onto the PLGA membranes by PECVD in a plasma reactor with a remote configuration. The deposition system consisted of a quartz tube with a funnel shape termination attached to a stainless-steel chamber where the samples were placed. The plasma source is a surfatron (Sairem) launcher that was supplied with a microwave power of 60 W. These conditions provide a typical plasma electron density of about 1 · 10^11^ cm^−3^. Distance from substrate to the quartz tube was 5 cm. The plasma gas and the precursor used were pure Ar (7.5 sccm) and hexamethyldisiloxane (HDMSO, 5 sccm), respectively. Synthesis of the films was carried out at room temperature, working at a pressure of 0.3 Torr. The gas flow was controlled by mass flow controllers (MKS). Both the stainless steel receptacle which contained the precursor and the dosing line were heated at 323 K to prevent any condensation in the tube walls. Before depositing SiO_2_, the polymeric substrate was exposed to plasma of pure argon for 2 min, a pretreatment that favours the adhesion of the oxide layer onto the polymer surface. A thin layer of SiO_2_ of 15 nm, determined by measuring with an adjacent quartz crystal monitor, was deposited on the polymeric substrate. These measurements were calibrated with the data obtained for thicker layer of SiO_2_ deposited on a silicon substrate that were cleaved and examined by scanning electron microscopy. Before the treatments, Ar or the mixture Ar + HDMSO was kept flowing for at least 30 min to ensure the purity of the plasma gas in the reactor [36]. The nonfunctionalized and SiO_2_ functionalized membranes will be designated as PLGA and SiO_2_/PLGA, respectively. The samples were characterized by X-ray photoelectron spectroscopy (XPS) to determine their surface chemical state and composition, atomic force microscopy (AFM) to ascertain their roughness, scanning electron microscopy (SEM) to assess the film microstructure, infrared spectroscopy (FT-IR) to determine the bonding structure, and water contact angle measurements to check the wetting characteristics of their surfaces. XPS spectra were recorded with a SPECS PHOIBOS-100 spectrometer working in the constant pass energy mode fixed at a value of 20 eV. The Mg K_a_ radiation was used as excitation source. For calibration of  the binding energy scale, a value of 284.6 eV was considered for the C1s component attributed to C–H and C–C bonds. Surface composition was estimated by calculating the area behind the C_1s_, Si_2p_, and O_1s_ peaks and by correcting the obtained values with the sensitivity factors of these elements. AFM images were taken with a Cervantes AFM microscope driven with a Dulcinea control system (Nanotec, Madrid, Spain) working in tapping mode and using high frequency cantilevers. The images were processed with Nanotec WSxM software. SEM images were acquired in a Hitachi S4800 microscope. FT-IR spectra were collected in attenuated total reflectance (ATR) mode in a JASCO FT/IR6200 spectrometer, performing 64 scans with a resolution of 0.96 cm^−1^ and a spot size of 1 cm of diameter. Static contact angles of water were determined by the Young method in a SCA20 instrument (Data Physic Instruments, GmbH), with an estimated error of 3%.

### 2.4. Cell Morphology and Spreading

HOB osteoblasts seeded on test surfaces were daily examined with a DM ILLED Leica phase contrast microscope in order to evaluate cell morphology and spreading, cell alignment, and initial adhesion phase to surfaces, prior to fluorescence and CLSM examination.

### 2.5. Immunolabelling for Actin Cytoskeletal Organization and Focal Adhesion Expression

At the designed experimental time, cells in each group were washed with prewarmed phosphate buffered saline (PBS) and fixed with 3.7% paraformaldehyde at room temperature, permeabilized with 0.1% Triton x-100, preincubated in 1% bovine serum albumin in PBS, and then immunolabelled for actin cytoskeleton with rhodamine-phalloidin and for focal adhesion identification with monoclonal antivinculin FITC conjugate. After 20 min samples were twice PBS rinsed prior to mounting with Vectashield (Vector, CA). All chemicals were obtained from Sigma-Aldrich (St. Louis, Mo) unless noted.

### 2.6. Immunolabelled Cells Examination

Samples were visualized in a Leica TCS-SL confocal microscope. At least five samples were analysed for each experimental group to assess surface influence on cytoskeletal organization, focal adhesion number and development, and cell morphology. Samples were exposed to the lowest laser power that was able to produce a fluorescent signal, with a pinhole of 1 airy. Images were acquired at a resolution of 512 × 512, mean voxel size of 209.20 nm.

### 2.7. Image Analysis

To analyze the differences in focal adhesion number between different sample groups, images were collected and processed. Area, perimeter, roundness, circularity, solidity, and axial ratio were analysed as shape variables. Sample images were collected as frames obtained at 40x magnification and processed using Leica imaging software and Image J software (NIH, http://rsb.info.nih.gov/ij/).

### 2.8. Statistical Analysis

For the variable number of contacts, we used first the nonparametric contrast Kruskal-Wallis and later a two-way ANOVA contrast with the ranges of this variable [37]. Post hoc contrasts were carried out to detect the differences among the experimental groups. For shape variables, a factor analysis was performed in order to reduce the dimension and a two-way ANOVA with the resulting factors to analyze the influence of the experimental conditions. All the analysis was performed with SPSS program.

## 3. Results

### 3.1. PLGA Membranes: Deposition and Characterization of SiO_2_ Thin Film Layers

**Figure 1:**
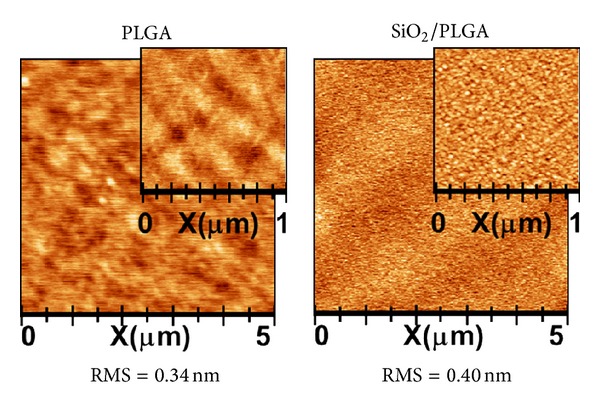
AFM images of PLGA (left) and SiO_2_/PLGA (right).

**Figure 2:**
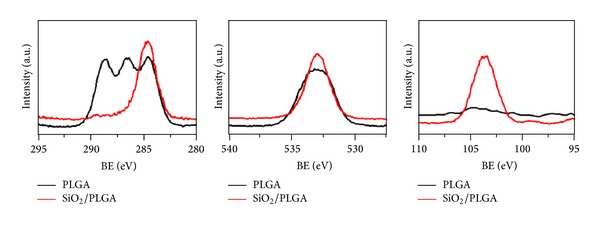
From left to right, C1s, O1s, and Si2p photoelectron spectra of PLGA and SiO_2_/PLGA as indicated.

The SiO_2_ thin films prepared by PECVD presented a homogeneous microstructure and a conformal growth on the PLGA membrane. The 15 nm film surface was rather flat as shown in AFM images (Figure 1) of PLGA and SiO_2_-PLGA samples. In the latter, the deposition of the SiO_2_ film only increased the RMS roughness from 0.32 to 0.43 nm, while surface topology slightly changed with the formation of finer grains. Even if the SiO_2_ thickness was rather thin (15 nm), the PLGA surface was totally covered by SiO_2_, as can be directly retrieved from the XPS spectra (Figure 2).

**Figure 3:**
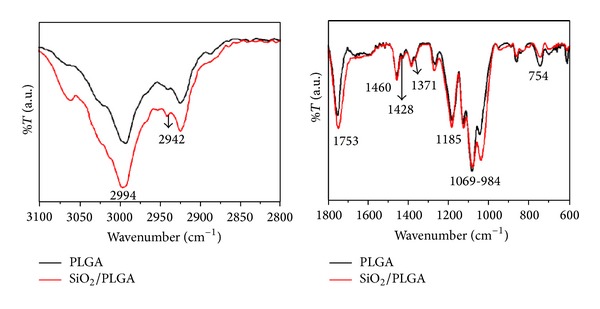
FT-IR spectra of the PLGA and SiO_2_/PLGA films measured in ATR mode.

The C1s spectrum of the PLGA depicts three components at 284.6, 286.4, and 288.6 eV, attributed to C–C/C–H, C–O, and >C=O/COOH species [22, 23]. This complex spectrum transformed when the PLGA membrane was covered by SiO_2_, where only a C1s component at 284.6 eV, attributed to spurious carbon due to contamination, can be identified. An equivalent modification appears in the O1s spectra, with clearly different widths of PLGA and SiO_2_ spectra. The Si2p spectra confirms that the PLGA membrane was free from this element, while the SiO_2_/PLGA samples depict a well-defined Si 2p peak at 103.9 eV, attributed to Si^4+^ ions in SiO_2_ [38, 39]. It must be stressed that the deposition by PECVD of the SiO_2_ onto the PLGA membrane did not alter the composition and other characteristics of the organic membrane as proved by the FT-IR analysis of samples. The spectral regions reported (Figure 3) for the PLGA and SiO_2_-PLGA samples were very similar and are characterized by the typical shape of the spectra of this polymer. The small increase in the height of the peak at 1050 cm^−1^ observed for the SiO_2_/PLGA sample can be attributed to the contribution at this wavenumbers of the Si–O–Si stretching vibration of the silica network in the SiO_2_ film [36]. The other features in the spectra correspond to well-documented vibrations of PLGA polymers: 2994, 2946, 2840 (CH bend), 1753 (C=O ester), 1460, 1428, 1371 (CH_3_), 1185, 1080–980 (C–O stretch), and 754 (CH-bend) cm^−1^ [40]. No significant spectral changes are expected due to the different thickness of the PLGA membrane (50 *μ*m) and the SiO_2_ films (15 nm) and the typical thickness proved by ATR analysis (a few tens of microns).

Since wetting behavior and surface tension are important parameters for cell development of cells on bioactive materials [41, 42] the water contact angle was measured for both the PLGA and SiO_2_/PLGA membranes and the values obtained were 99.3° and 93.8°, respectively, indicating no significant differences in wetting between these two surfaces.

During the experimental time, no clear signs of degradation were observed in the membranes, During the experimental time, no clear signs of degradation were observed in the membranes. FT-IR, AFM, and XPS analysis of the surface of the PLGA and SiO_2_/PLGA membranes extracted from the PBS and gentled dried in air did not reveal significant changes suggesting any significant degradation. membrane degradation (loss of physical integrity, changes in color, etc.) could only be observed after 20 weeks of immersion in liquid media, although clear signs of degradation (loss of physical integrity, changes in color, etc.) could be observed after 20 weeks of immersion in liquid media. The exposure of PLGA and SiO_2_/PLGA membranes to u.v. irradiation under similar conditions to those used in the sterilization protocol prior to cell seeding did not produce any detectable change.

### 3.2. Living Cell Examination: Cell Morphology and Spreading

**Figure 4:**
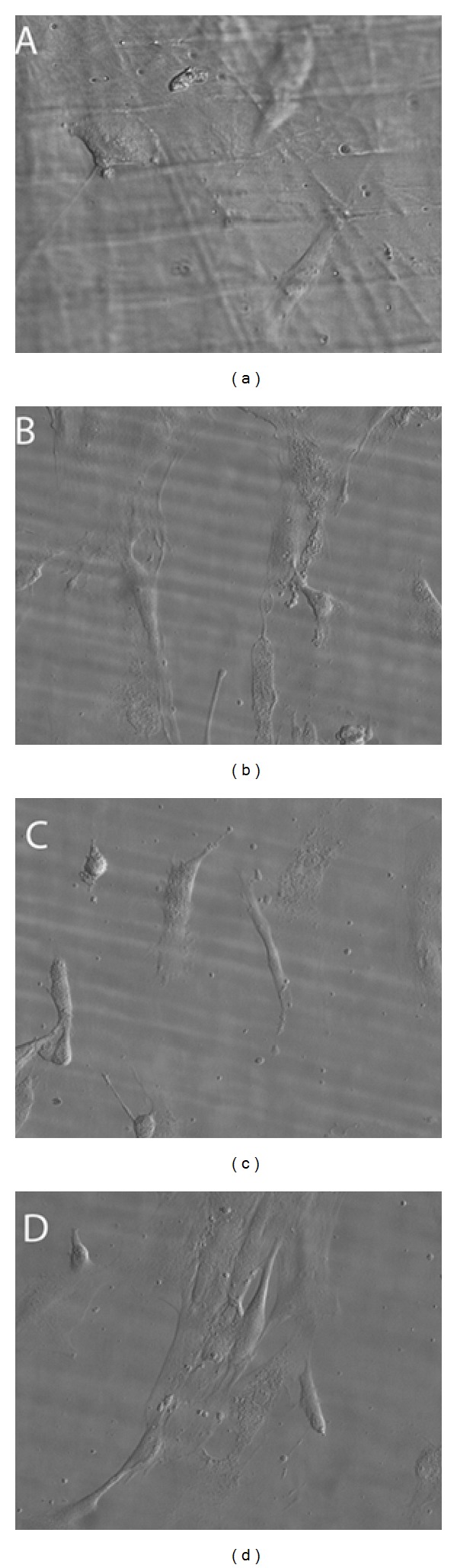
HOB cells distribution and spreading after 24 (a, b) and 48 (c, d) hours in culture. Both (b) and (d) represent osteoblasts grown on silica treated PLGA membranes and (a) and (c) represent osteoblasts grown on bare PLGA. All images were obtained in the phase contrast microscope, magnification 40x.

Living HOB  cells examination revealed a successful cell spreading on silica treated membranes, with cell elongation and marked filopodial emission, and the establishment of clear cell contacts and attachment to the functionalized surface in the first 24 hours. Cells clustered and elongated during the next hours, and after 48 hours in culture, cell spreading evolved to a clear cell packaging, with osteoblasts well adhered to the silica treated surface and to the neighboring cells (Figures 4(b) and 4(d)). Cells grown on bare PLGA membranes spread evenly although clearly adhered to surfaces, showing some lamellipodia in the first 24 hours but without clear cell clustering after 48 hours in culture (Figures 4(a) and 4(c)).

### 3.3. Actin Cytoskeletal Organization and Vinculin Immunolabelling of Focal Adhesion Sites

**Figure 5:**
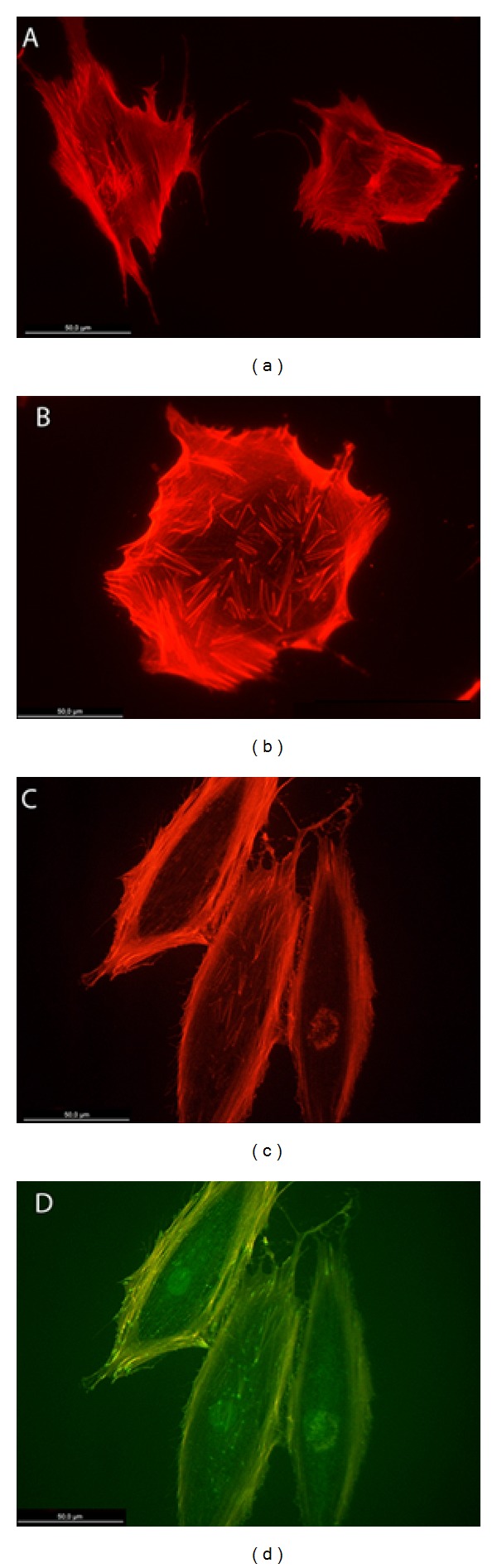
HOB cells grown on bare PLGA and imunolabelled, 48 hours (a, b) and 72 hours (c, d) after seeding, with rhodamine-phalloidin for actin cytoskeleton (red) and antivinculin antibody (green) for focal adhesion sites.

**Figure 6:**
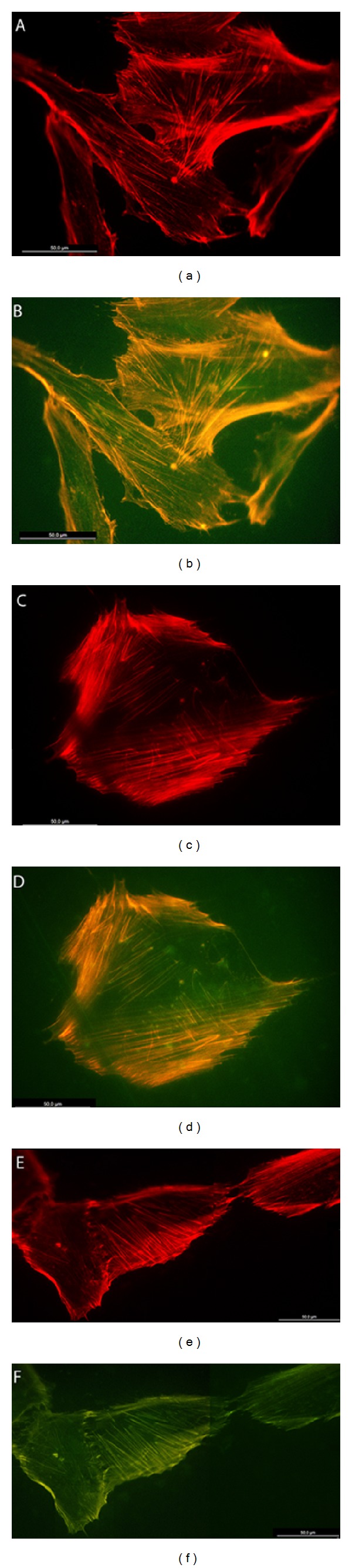
HOB cells grown on SiO_2_ functionalized membranes, immunolabelled with rhodamine-phalloidin for actin cytoskeleton (red) and antivinculin antibody (green) for focal adhesion sites, 48 hours after seeding. Representative images of cytoskeletal features and focal adhesions, showing in detail (a, b) stress fibers development, (c, d) cell clustering, and (e, f) lamellipodial and (g, h) filopodial emissions.

**Figure 7:**
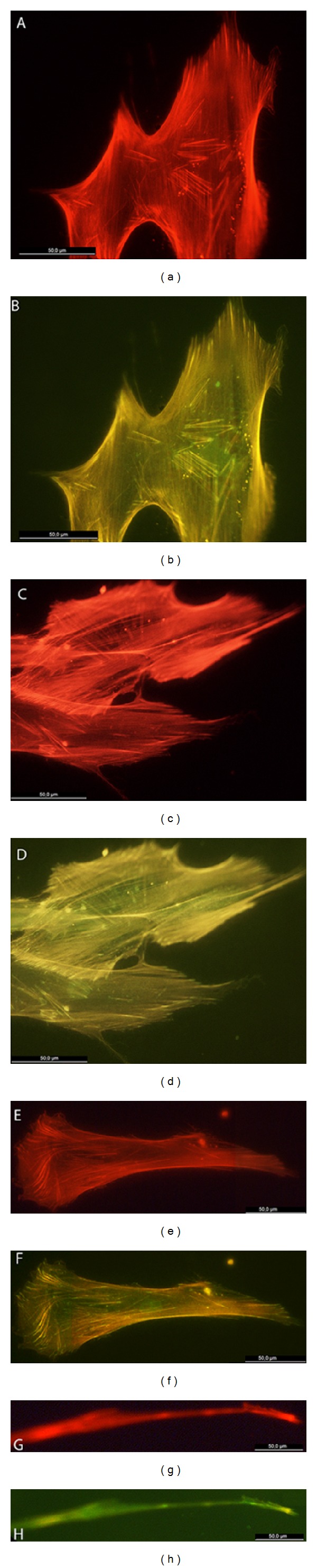
HOB cells grown on SiO_2_ functionalized membranes, immunolabelled with rhodamine-phalloidin for actin cytoskeleton (red) and antivinculin antibody (green) for focal adhesion sites, 72 hours after seeding. Representative images for (a, b, e, f) cell clustering, (c, d) stress fibers distribution, and (e, f) lamellipodial emissions.

Osteoblasts grown on the SiO_2_ functionalized PLGA membranes showed defined stress fibers formation, with a clear polarization of actin cytoskeleton towards strongly evident vinculin positive sites. Cell polarization was clearly determined by SiO_2_ functionalized surfaces, with osteoblasts showing extensive lamellipodial emission reinforced with vinculin positive focal adhesion in the leading edge and supported by a highly defined actin network. Focal adhesions appeared to be significantly more numerous and well developed in those cells grown on the SiO_2_ functionalized membranes HOB grown on bare PLGA randomly orientated and showed a reduced stress fibers and focal adhesion development at the same experimental time. Although some evenly distributed filopodia were observed, cells did not show a well-organized actin network or polarization, and vinculin positive sites appeared randomly distributed in the cell periphery (Figures 5, 6, and 7).

### 3.4. Image Analysis: Statistical Analysis of Focal Adhesion Behaviour

**Figure 8:**
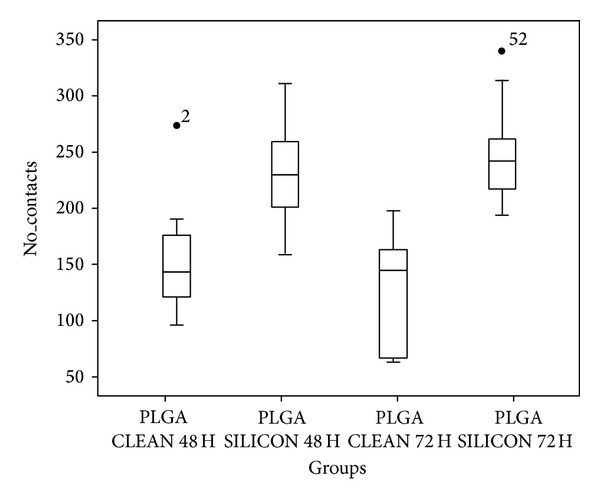
Box-Whisker graphics reporting the variable number of contacts among cells on each sample.

The high significance obtained after the quantitative analysis of focal adhesion number by Kruskal-Wallis nonparametric contrast for the 4 groups obtained with the combination of the factors* silicon* and* time* (*H* = 23.506, *P* = 0.000, df = 3) indicates that silicon, time, or both influence focal adhesion number. Using the two-way ANOVA, the analysis of the effects of each factor and their interaction with the ranks of the variable [38], our results indicate that the model is able to explain 58.8% and that* silicon* is a significant factor (*P* = 0.000), while* time* factor is not (*P* = 0.929). Furthermore, silicon treated groups (post hoc contrast of Scheffé, *P* = 0.819) and untreated groups (*P* = 0.873) can be considered homogeneous at any time, and both groups are different from each other (*P* = 0.05) (Figure 8, Table 1).

### 3.5. Image Analysis: Statistical Analysis of the Shape Variables

The descriptive analysis of the shape variables in the osteoblasts grown in the four experimental groups did not establish a clear relationship with the factors* silicon* or* time*. Then, an exploratory factor analysis was performed, in the attempt to reduce the number of morphometric indexes (latent factors). The correlation matrix and the factor loadings matrix were obtained first by the method of principal components and later by varimax rotation, which quantify the relationship between the original variables and the indexes achieved. The first index positively related to* circularity* and* solidity* and negatively related to* perimeter*. This first index represents the property of* solid roundness* (SR) showing higher values in those cells that are circular, without voids, and with a medium perimeter. The second index contrasts* roundness* and AR, thus representing* stretch* (ST) or* lengthening*, with the highest values for the more elongated cells, with high AR, and negative values more circular cells, with AR close to 1. The third index relates to* area* and* perimeter* and can, therefore, be interpreted as the cell* size*, with higher values for larger cells.

Growing cells were scored for the morphometric indices according to the values obtained in each one by each individual cell. The two-way ANOVA contrast performed for these indices showed that the experimental factor* silicon* significantly increased (*P* = 0.000) the mean score for SR index, estimating this increase by CI95% = (0.94, 1.71), and also caused a decrease in ST index (*P* = 0.016), estimated by CI95% = (−1.03, −0.11). Moreover, the factor* time* caused a decrease in the average score for the ST index (*P* = 0.012), estimated by CI95% = (−1.06, −0.14), while a significant interaction (*P* = 0.029) was obtained for the index* size*, as cell* size* decreases in cells grown on bare PLGA surfaces, CI95% = (−1.28, 0.65), and increases in silicon treated ones, CI95% = (0.32, 1.30). These results indicate that silica treatment, independently of time, favors the presence of cells that were* circular* and* solid* (without voids). On the other hand, and independently of silica treatment, the experimental factor* time* favors a lower ST, or* roundness*, and increases the* size* in cells grown on silica treated membranes.

## 4. Discussion

There are several ways to modify the surface of a material in order to enhance its attractiveness for cell colonization and osteogenic cell differentiation. For biological applications, there is an additional need for versatile deposition methods in the case of devices with potential clinical applications, which should also stand, without alterations, the sterilization processes [13, 16, 18]. We have successfully demonstrated that PECVD TiO_2_ functionalization methodology employed by our group enhances HOB cells response to nonresorbable PET devices and stands for standard u.v. sterilization [43]. We now herein present a novel design of barrier membrane for GBR purposes, in which HOB cells response to PECVD SiO_2_ functionalized PLGA membranes has been assessed.

Trace amounts of silicon have been described to substantially contribute to strengthening of bones with promotion of bone formation especially during ageing. Well-dispersed nanosized inorganic particles have been described to enhance the mechanical properties of organic materials [10]. A recent study on biosilica influence on the OPG-RANKL ratio in SaOS-2 cells in vitro suggests both osteoinductive and osteogenic potential of biosilica since it combines an upregulation of the genes required for the differentiation with the potential to increase proliferation of the osteogenic cells [18, 24].

Our PLGA membranes were fabricated by using a conventional resorbable PLGA polymer as substrate and then functionalized by depositing a very thin layer of SiO_2_, as active layer, by PECVD. A key feature of this method is that it preserved the integrity of the substrate, which remained practically unaltered after the SiO_2_ as revealed by FT-IR analysis (Figure 3). In comparison with wet chemical routes, which may affect the integrity of this sensitivity substrate, PECVD has been demonstrated to be a quite controllable and friendly procedure for the surface functionalization of resorbable polymers. The absence of a significant damage has been proved here by the AFM analysis of the SiO_2_/PLGA substrate showing that the surface roughness is not significantly altered after the deposition of SiO_2_. Stability of these membranes after 72 hours of immersion in a PBS liquid and after UV irradiation sustains that the in vitro cell growth analysis basically refers to the “as prepared” samples without any significant alteration during its manipulation or during their immersion in the culture medium.

Cell adhesion onto a biomaterial surface is a major contributing factor of its cytocompatibility and the host response when implanted, ultimately determining device biocompatibility. Without adhesion, osteoblasts cannot spread or differentiate. A* princeps* issue in cell adhesion is the development of focal adhesions which, furthermore, are established sites for signal transduction, acting as conveyors of mechanical forces directly to the nucleus via the cytoskeletal network by the complex process of mechanotransduction, where vinculin is required for a strong network formation [1, 4, 44]. Based on the fact that the size and shape of cell spreading area, as well as the number and distribution of  focal adhesion plaques, are decisive for migratory, proliferative, and differentiation behaviour of anchorage-dependent cells, we performed animage-based quantitative feature extraction design in which focal adhesion quantification has been used as marker to assess the osteoconductive potential of the substrata, together with actin cytoskeleton arrangement [2, 17, 20, 45].

The relation between focal adhesion number, functionality, cell movement, and growth of skeletal cells has been described, by our group and others [46], and the formation of super-mature adhesions has also been related to osteogenesis support [47]. Actin polymerization and cytoskeleton rearrangement induced by topographical and chemical cues present in the growing surface are known to serve as driving forces for directional migration and morphological alterations. Of particular interest is the temporal and spatial reorganization of cytoskeleton and the focal adhesion formation in response to SiO_2_ layer identified in our model, parameters that have been established as important mediators of the mechanotransductive processes, parameters that have been established as important mediators of the mechanotransductive processes [17, 47, 48].

Adherent cells require extracellular matrix anchorage to proliferate and undergo differential function. As shown by our data, HOB cells grown on SiO_2_ functionalized PLGA membranes actively probe the physical properties of the ECM, with their contractile machinery clearly facilitating the formation of polarized lamellipodia and evenly distributed filopodia, which gather spatial, topographical, and chemical information from the surface. Initial cell tethering and filopodia exploration are followed by lamellipodia ruffling membrane activity and cellular spreading. The results, obtained after a carefully designed in vitro study, are reinforced with a powerful statistical treatment and reveal that in vitro HOB cell morphologies are significantly different and vary greatly depending on the substrata and are consistent with an increased cytoskeletal tension related to focal adhesion increase in SiO_2_ treated membranes.

## 5. Conclusions

The methodology described provides a biocompatible and durable functionalization on our PLGA barrier membranes that elicits a significant osteoblast response. Both the morphological changes and the focal adhesion development identified in our model significantly related to SiO_2_ treated PLGA both the morphological changes and the focal adhesion development identified in our model are significantly related to SiO_2_ treated PLGA. The novedous SiO_2_ deposition method reveals as a valuable tool to increase bioactivity of PLGA membranes by combining cell nanotopography cues with the incorporation of bioactive factors, that in addition, stands the standard sterilization protocols.

## Figures and Tables

**Table 1:** Variable number of contacts. Descriptive parameters.

	Groups	*N *	Minimum	Maximum	Median	Mean	St. dev.
1	PLGA CLEAN 48 H	11	96	274	143	154.27	49.601
2	PLGA SILICON 48 H	11	159	311	229	230.73	46.866
3	PLGA CLEAN 72 H	9	63	198	145	128.89	51.163
4	PLGA SILICON 72 H	10	194	340	241.5	250.50	46.049
